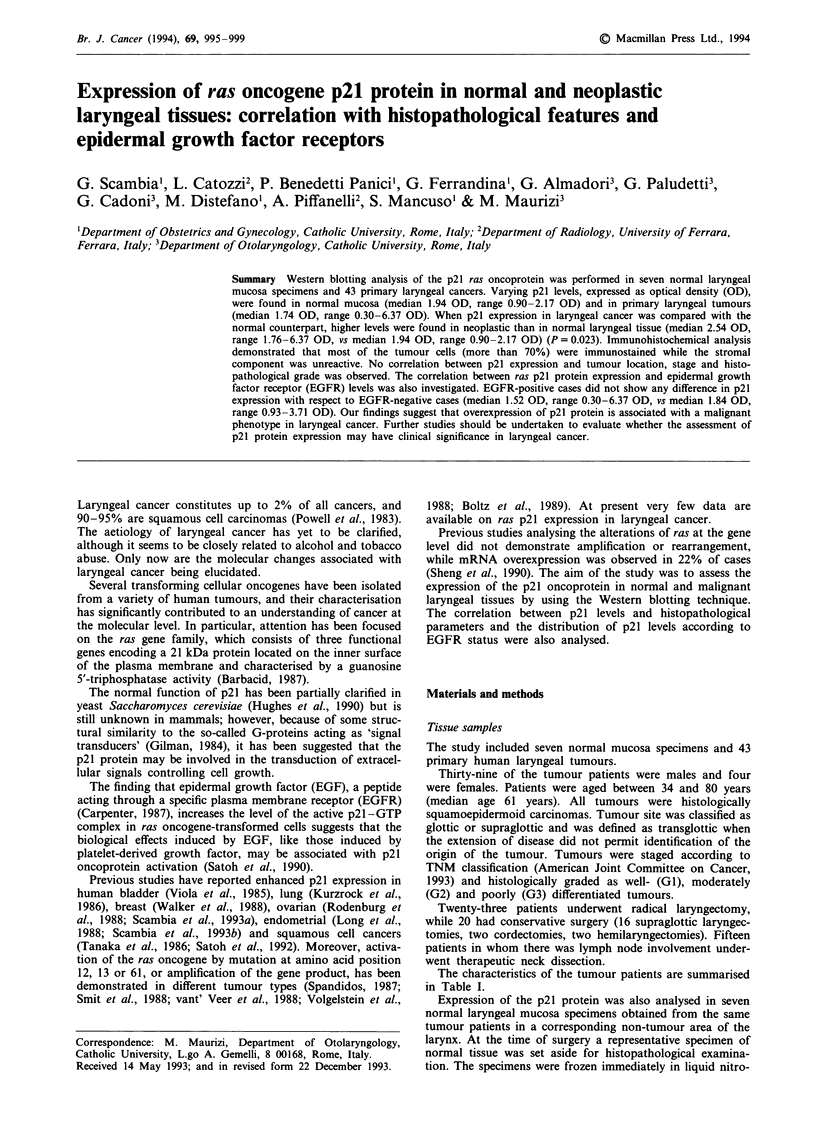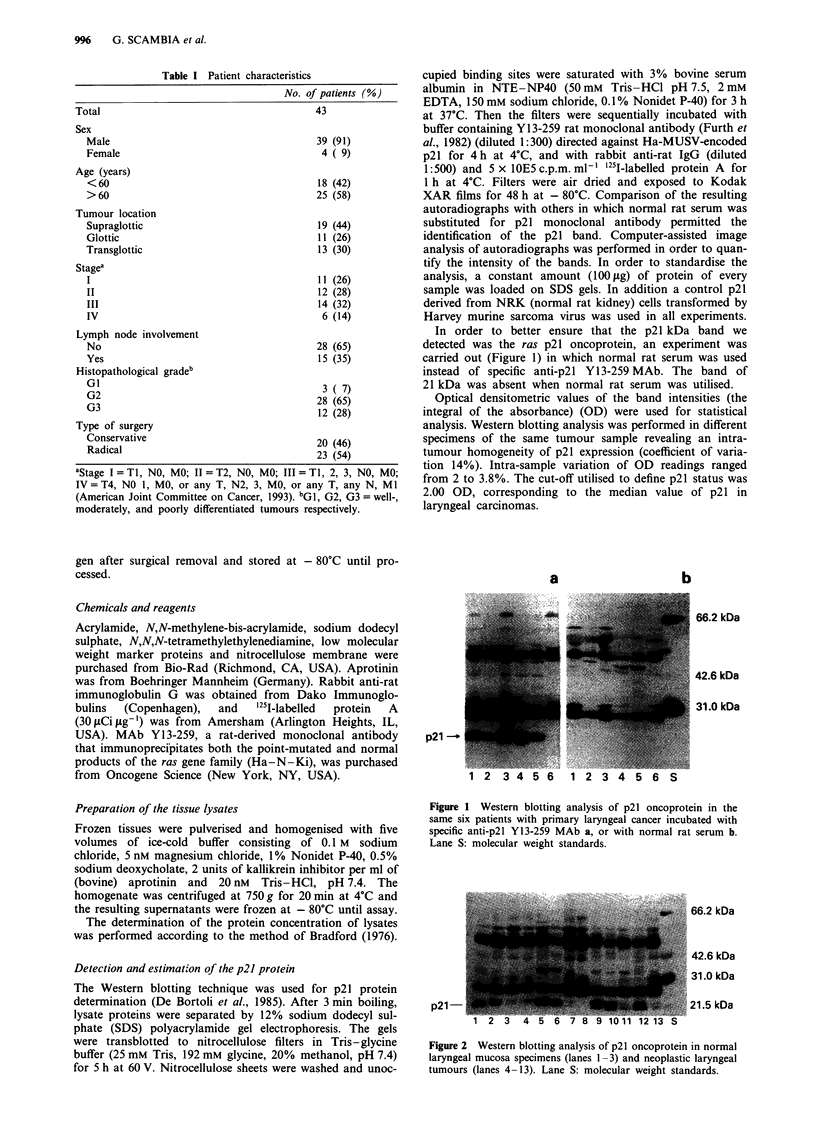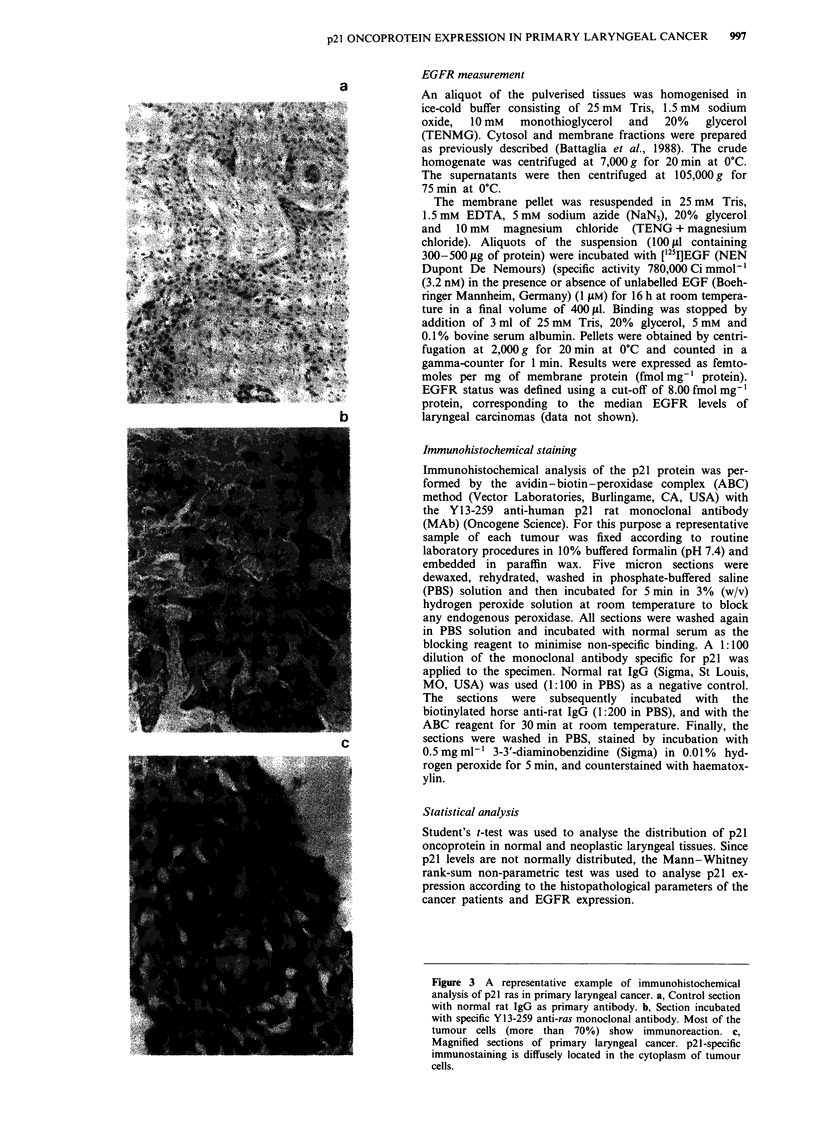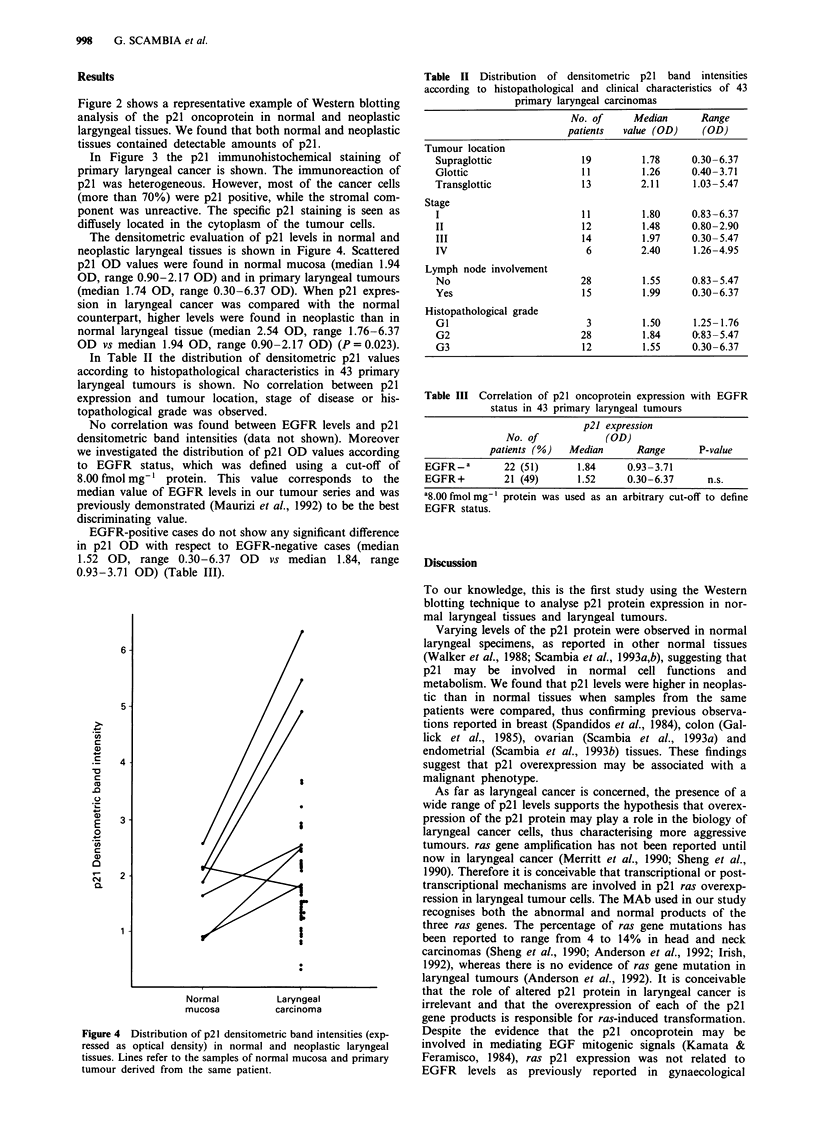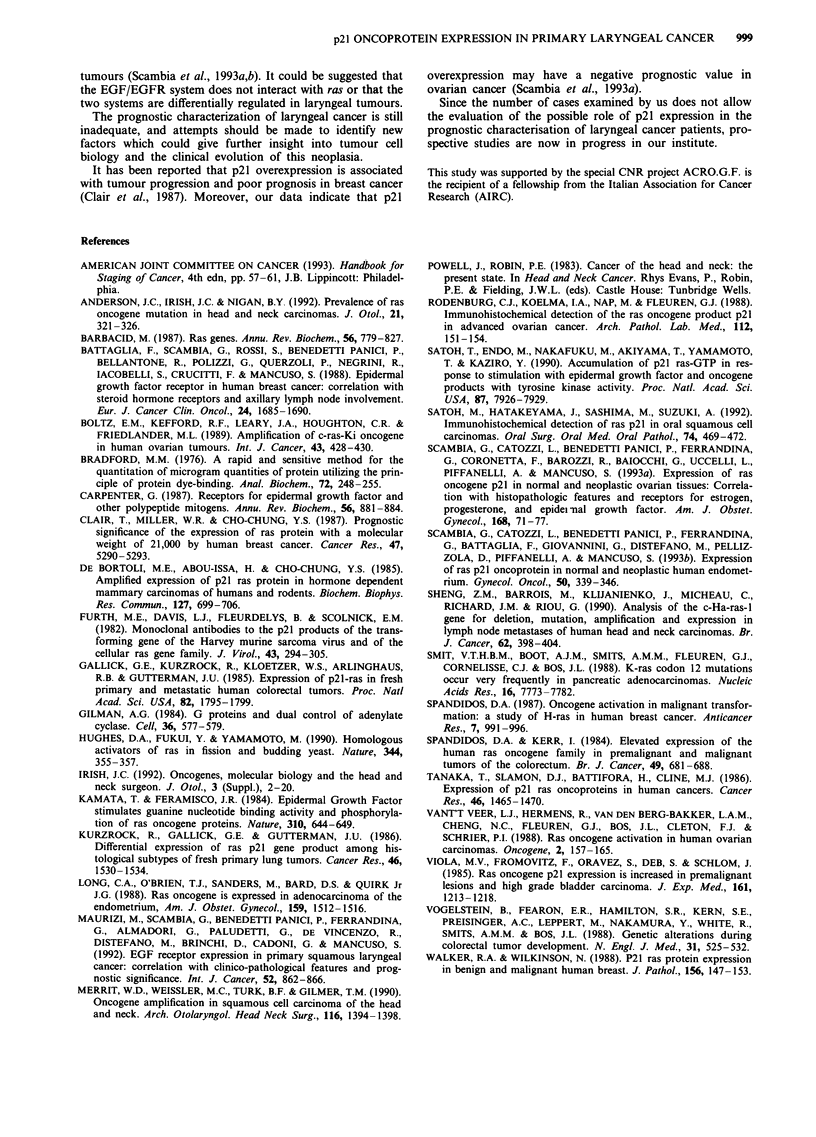# Expression of ras oncogene p21 protein in normal and neoplastic laryngeal tissues: correlation with histopathological features and epidermal growth factor receptors.

**DOI:** 10.1038/bjc.1994.195

**Published:** 1994-06

**Authors:** G. Scambia, L. Catozzi, P. Benedetti Panici, G. Ferrandina, G. Almadori, G. Paludetti, G. Cadoni, M. Distefano, A. Piffanelli, S. Mancuso

**Affiliations:** Department of Obstetrics and Gynecology, Catholic University, Rome, Italy.

## Abstract

**Images:**


Br. J. Cancer (1994), 69, 995–999 © Macmillan Press Ltd., 1994

### Expression of *ras* oncogene p21 protein in normal and neoplastic laryngeal tissues: correlation with histopathological features and epidermal growth factor receptors

G. Scambia[1], L. Catozzi[2], P. Benedetti Panici[1], G. Ferrandina[1], G. Almadori[3], G. Paludetti[3], G. Cadoni[3], M. Distefano[1], A. Piffanelli[2], S. Mancuso[1] & M. Maurizi[3]

[1]*Department of Obstetrics and Gynecology, Catholic University, Rome, Italy;* [2]*Department of Radiology, University of Ferrara, Ferrara, Italy;* [3]*Department of Otolaryngology, Catholic University, Rome, Italy*

**Summary** Western blotting analysis of the p21 *ras* oncoprotein was performed in seven normal laryngeal mucosa specimens and 43 primary laryngeal cancers. Varying p21 levels, expressed as optical density (OD), were found in normal mucosa (median 1.94 OD, range 0.90–2.17 OD) and in primary laryngeal tumours (median 1.74 OD, range 0.30–6.37 OD). When p21 expression in laryngeal cancer was compared with the normal counterpart, higher levels were found in neoplastic than in normal laryngeal tissue (median 2.54 OD, range 1.76–6.37 OD, *vs* median 1.94 OD, range 0.90–2.17 OD) ($P = 0.023$). Immunohistochemical analysis demonstrated that most of the tumour cells (more than 70%) were immunostained while the stromal component was unreactive. No correlation between p21 expression and tumour location, stage and histopathological grade was observed. The correlation between *ras* p21 protein expression and epidermal growth factor receptor (EGFR) levels was also investigated. EGFR-positive cases did not show any difference in p21 expression with respect to EGFR-negative cases (median 1.52 OD, range 0.30–6.37 OD, *vs* median 1.84 OD, range 0.93–3.71 OD). Our findings suggest that overexpression of p21 protein is associated with a malignant phenotype in laryngeal cancer. Further studies should be undertaken to evaluate whether the assessment of p21 protein expression may have clinical significance in laryngeal cancer.

Laryngeal cancer constitutes up to 2% of all cancers, and 90–95% are squamous cell carcinomas (Powell *et al.*, 1983). The aetiology of laryngeal cancer has yet to be clarified, although it seems to be closely related to alcohol and tobacco abuse. Only now are the molecular changes associated with laryngeal cancer being elucidated.

Several transforming cellular oncogenes have been isolated from a variety of human tumours, and their characterisation has significantly contributed to an understanding of cancer at the molecular level. In particular, attention has been focused on the *ras* gene family, which consists of three functional genes encoding a 21 kDa protein located on the inner surface of the plasma membrane and characterised by a guanosine 5′-triphosphatase activity (Barbacid, 1987).

The normal function of p21 has been partially clarified in yeast *Saccharomyces cerevisiae* (Hughes *et al.*, 1990) but is still unknown in mammals; however, because of some structural similarity to the so-called G-proteins acting as 'signal transducers' (Gilman, 1984), it has been suggested that the p21 protein may be involved in the transduction of extracellular signals controlling cell growth.

The finding that epidermal growth factor (EGF), a peptide acting through a specific plasma membrane receptor (EGFR) (Carpenter, 1987), increases the level of the active p21–GTP complex in *ras* oncogene-transformed cells suggests that the biological effects induced by EGF, like those induced by platelet-derived growth factor, may be associated with p21 oncoprotein activation (Satoh *et al.*, 1990).

Previous studies have reported enhanced p21 expression in human bladder (Viola *et al.*, 1985), lung (Kurzrock *et al.*, 1986), breast (Walker *et al.*, 1988), ovarian (Rodenburg *et al.*, 1988; Scambia *et al.*, 1993*a*), endometrial (Long *et al.*, 1988; Scambia *et al.*, 1993*b*) and squamous cell cancers (Tanaka *et al.*, 1986; Satoh *et al.*, 1992). Moreover, activation of the *ras* oncogene by mutation at amino acid position 12, 13 or 61, or amplification of the gene product, has been demonstrated in different tumour types (Spandidos, 1987; Smit *et al.*, 1988; vant' Veer *et al.*, 1988; Volgelstein *et al.*, 1988; Boltz *et al.*, 1989). At present very few data are available on *ras* p21 expression in laryngeal cancer.

Previous studies analysing the alterations of *ras* at the gene level did not demonstrate amplification or rearrangement, while mRNA overexpression was observed in 22% of cases (Sheng *et al.*, 1990). The aim of the study was to assess the expression of the p21 oncoprotein in normal and malignant laryngeal tissues by using the Western blotting technique. The correlation between p21 levels and histopathological parameters and the distribution of p21 levels according to EGFR status were also analysed.

### Materials and methods

### *Tissue samples*

The study included seven normal mucosa specimens and 43 primary human laryngeal tumours.

Thirty-nine of the tumour patients were males and four were females. Patients were aged between 34 and 80 years (median age 61 years). All tumours were histologically squamoepidermoid carcinomas. Tumour site was classified as glottic or supraglottic and was defined as transglottic when the extension of disease did not permit identification of the origin of the tumour. Tumours were staged according to TNM classification (American Joint Committee on Cancer, 1993) and histologically graded as well- (G1), moderately (G2) and poorly (G3) differentiated tumours.

Twenty-three patients underwent radical laryngectomy, while 20 had conservative surgery (16 supraglottic laryngectomies, two cordectomies, two hemilaryngectomies). Fifteen patients in whom there was lymph node involvement underwent therapeutic neck dissection.

The characteristics of the tumour patients are summarised in Table I.

Expression of the p21 protein was also analysed in seven normal laryngeal mucosa specimens obtained from the same tumour patients in a corresponding non-tumour area of the larynx. At the time of surgery a representative specimen of normal tissue was set aside for histopathological examination. The specimens were frozen immediately in liquid nitro-

Correspondence: M. Maurizi, Department of Otolaryngology, Catholic University, L.go A. Gemelli, 8 00168, Rome, Italy.
Received 14 May 1993; and in revised form 22 December 1993.

**Table I** Patient characteristics

| | No. of patients (%) |
|---|---|
| Total | 43 |
| Sex | |
| Male | 39 (91) |
| Female | 4 ( 9) |
| Age (years) | |
| <60 | 18 (42) |
| >60 | 25 (58) |
| Tumour location | |
| Supraglottic | 19 (44) |
| Glottic | 11 (26) |
| Transglottic | 13 (30) |
| Stage[a] | |
| I | 11 (26) |
| II | 12 (28) |
| III | 14 (32) |
| IV | 6 (14) |
| Lymph node involvement | |
| No | 28 (65) |
| Yes | 15 (35) |
| Histopathological grade[b] | |
| G1 | 3 ( 7) |
| G2 | 28 (65) |
| G3 | 12 (28) |
| Type of surgery | |
| Conservative | 20 (46) |
| Radical | 23 (54) |

[a]Stage I = T1, N0, M0; II = T2, N0, M0; III = T1, 2, 3, N0, M0; IV = T4, N0 1, M0, or any T, N2, 3, M0, or any T, any N, M1 (American Joint Committee on Cancer, 1993). [b]G1, G2, G3 = well-, moderately, and poorly differentiated tumours respectively.

gen after surgical removal and stored at − 80°C until processed.

### *Chemicals and reagents*

Acrylamide, *N,N*-methylene-bis-acrylamide, sodium dodecyl sulphate, *N,N,N*-tetramethylethylenediamine, low molecular weight marker proteins and nitrocellulose membrane were purchased from Bio-Rad (Richmond, CA, USA). Aprotinin was from Boehringer Mannheim (Germany). Rabbit anti-rat immunoglobulin G was obtained from Dako Immunoglobulins (Copenhagen), and $^{125}$I-labelled protein A (30 $\mu$Ci $\mu g^{-1}$) was from Amersham (Arlington Heights, IL, USA). MAb Y13-259, a rat-derived monoclonal antibody that immunoprecipitates both the point-mutated and normal products of the *ras* gene family (Ha-N-Ki), was purchased from Oncogene Science (New York, NY, USA).

### *Preparation of the tissue lysates*

Frozen tissues were pulverised and homogenised with five volumes of ice-cold buffer consisting of 0.1 M sodium chloride, 5 nM magnesium chloride, 1% Nonidet P-40, 0.5% sodium deoxycholate, 2 units of kallikrein inhibitor per ml of (bovine) aprotinin and 20 nM Tris-HCl, pH 7.4. The homogenate was centrifuged at 750 *g* for 20 min at 4°C and the resulting supernatants were frozen at − 80°C until assay.

The determination of the protein concentration of lysates was performed according to the method of Bradford (1976).

### *Detection and estimation of the p21 protein*

The Western blotting technique was used for p21 protein determination (De Bortoli *et al.*, 1985). After 3 min boiling, lysate proteins were separated by 12% sodium dodecyl sulphate (SDS) polyacrylamide gel electrophoresis. The gels were transblotted to nitrocellulose filters in Tris-glycine buffer (25 mM Tris, 192 mM glycine, 20% methanol, pH 7.4) for 5 h at 60 V. Nitrocellulose sheets were washed and unoccupied binding sites were saturated with 3% bovine serum albumin in NTE-NP40 (50 mM Tris-HCl pH 7.5, 2 mM EDTA, 150 mM sodium chloride, 0.1% Nonidet P-40) for 3 h at 37°C. Then the filters were sequentially incubated with buffer containing Y13-259 rat monoclonal antibody (Furth *et al.*, 1982) (diluted 1:300) directed against Ha-MUSV-encoded p21 for 4 h at 4°C, and with rabbit anti-rat IgG (diluted 1:500) and 5 × 10E5 c.p.m. ml$^{-1}$ $^{125}$I-labelled protein A for 1 h at 4°C. Filters were air dried and exposed to Kodak XAR films for 48 h at − 80°C. Comparison of the resulting autoradiographs with others in which normal rat serum was substituted for p21 monoclonal antibody permitted the identification of the p21 band. Computer-assisted image analysis of autoradiographs was performed in order to quantify the intensity of the bands. In order to standardise the analysis, a constant amount (100 $\mu$g) of protein of every sample was loaded on SDS gels. In addition a control p21 derived from NRK (normal rat kidney) cells transformed by Harvey murine sarcoma virus was used in all experiments.

In order to better ensure that the p21 kDa band we detected was the *ras* p21 oncoprotein, an experiment was carried out (Figure 1) in which normal rat serum was used instead of specific anti-p21 Y13-259 MAb. The band of 21 kDa was absent when normal rat serum was utilised.

Optical densitometric values of the band intensities (the integral of the absorbance) (OD) were used for statistical analysis. Western blotting analysis was performed in different specimens of the same tumour sample revealing an intra-tumour homogeneity of p21 expression (coefficient of variation 14%). Intra-sample variation of OD readings ranged from 2 to 3.8%. The cut-off utilised to define p21 status was 2.00 OD, corresponding to the median value of p21 in laryngeal carcinomas.

**Figure 1** Western blotting analysis of p21 oncoprotein in the same six patients with primary laryngeal cancer incubated with specific anti-p21 Y13-259 MAb **a**, or with normal rat serum **b**. Lane S: molecular weight standards.

**Figure 2** Western blotting analysis of p21 oncoprotein in normal laryngeal mucosa specimens (lanes 1–3) and neoplastic laryngeal tumours (lanes 4–13). Lane S: molecular weight standards.

Figure 3 A representative example of immunohistochemical analysis of p21 ras in primary laryngeal cancer. **a**, Control section with normal rat IgG as primary antibody. **b**, Section incubated with specific Y13-259 anti-*ras* monoclonal antibody. Most of the tumour cells (more than 70%) show immunoreaction. **c**, Magnified sections of primary laryngeal cancer. p21-specific immunostaining is diffusely located in the cytoplasm of tumour cells.

### *EGFR measurement*

An aliquot of the pulverised tissues was homogenised in ice-cold buffer consisting of 25 mM Tris, 1.5 mM sodium oxide, 10 mM monothioglycerol and 20% glycerol (TENMG). Cytosol and membrane fractions were prepared as previously described (Battaglia *et al.*, 1988). The crude homogenate was centrifuged at 7,000 *g* for 20 min at 0°C. The supernatants were then centrifuged at 105,000 *g* for 75 min at 0°C.

The membrane pellet was resuspended in 25 mM Tris, 1.5 mM EDTA, 5 mM sodium azide ($NaN_3$), 20% glycerol and 10 mM magnesium chloride (TENG + magnesium chloride). Aliquots of the suspension (100 µl containing 300–500 µg of protein) were incubated with [$^{125}$I]EGF (NEN Dupont De Nemours) (specific activity 780,000 Ci mmol$^{-1}$ (3.2 nM) in the presence or absence of unlabelled EGF (Boehringer Mannheim, Germany) (1 µM) for 16 h at room temperature in a final volume of 400 µl. Binding was stopped by addition of 3 ml of 25 mM Tris, 20% glycerol, 5 mM and 0.1% bovine serum albumin. Pellets were obtained by centrifugation at 2,000 *g* for 20 min at 0°C and counted in a gamma-counter for 1 min. Results were expressed as femtomoles per mg of membrane protein (fmol mg$^{-1}$ protein). EGFR status was defined using a cut-off of 8.00 fmol mg$^{-1}$ protein, corresponding to the median EGFR levels of laryngeal carcinomas (data not shown).

### *Immunohistochemical staining*

Immunohistochemical analysis of the p21 protein was performed by the avidin–biotin–peroxidase complex (ABC) method (Vector Laboratories, Burlingame, CA, USA) with the Y13-259 anti-human p21 rat monoclonal antibody (MAb) (Oncogene Science). For this purpose a representative sample of each tumour was fixed according to routine laboratory procedures in 10% buffered formalin (pH 7.4) and embedded in paraffin wax. Five micron sections were dewaxed, rehydrated, washed in phosphate-buffered saline (PBS) solution and then incubated for 5 min in 3% (w/v) hydrogen peroxide solution at room temperature to block any endogenous peroxidase. All sections were washed again in PBS solution and incubated with normal serum as the blocking reagent to minimise non-specific binding. A 1:100 dilution of the monoclonal antibody specific for p21 was applied to the specimen. Normal rat IgG (Sigma, St Louis, MO, USA) was used (1:100 in PBS) as a negative control. The sections were subsequently incubated with the biotinylated horse anti-rat IgG (1:200 in PBS), and with the ABC reagent for 30 min at room temperature. Finally, the sections were washed in PBS, stained by incubation with 0.5 mg ml$^{-1}$ 3-3′-diaminobenzidine (Sigma) in 0.01% hydrogen peroxide for 5 min, and counterstained with haematoxylin.

### *Statistical analysis*

Student's *t*-test was used to analyse the distribution of p21 oncoprotein in normal and neoplastic laryngeal tissues. Since p21 levels are not normally distributed, the Mann–Whitney rank-sum non-parametric test was used to analyse p21 expression according to the histopathological parameters of the cancer patients and EGFR expression.

### Results

Figure 2 shows a representative example of Western blotting analysis of the p21 oncoprotein in normal and neoplastic largyngeal tissues. We found that both normal and neoplastic tissues contained detectable amounts of p21.

In Figure 3 the p21 immunohistochemical staining of primary laryngeal cancer is shown. The immunoreaction of p21 was heterogeneous. However, most of the cancer cells (more than 70%) were p21 positive, while the stromal component was unreactive. The specific p21 staining is seen as diffusely located in the cytoplasm of the tumour cells.

The densitometric evaluation of p21 levels in normal and neoplastic laryngeal tissues is shown in Figure 4. Scattered p21 OD values were found in normal mucosa (median 1.94 OD, range 0.90–2.17 OD) and in primary laryngeal tumours (median 1.74 OD, range 0.30–6.37 OD). When p21 expression in laryngeal cancer was compared with the normal counterpart, higher levels were found in neoplastic than in normal laryngeal tissue (median 2.54 OD, range 1.76–6.37 OD *vs* median 1.94 OD, range 0.90–2.17 OD) ($P = 0.023$).

In Table II the distribution of densitometric p21 values according to histopathological characteristics in 43 primary laryngeal tumours is shown. No correlation between p21 expression and tumour location, stage of disease or histopathological grade was observed.

No correlation was found between EGFR levels and p21 densitometric band intensities (data not shown). Moreover we investigated the distribution of p21 OD values according to EGFR status, which was defined using a cut-off of 8.00 fmol $mg^{-1}$ protein. This value corresponds to the median value of EGFR levels in our tumour series and was previously demonstrated (Maurizi *et al.*, 1992) to be the best discriminating value.

EGFR-positive cases do not show any significant difference in p21 OD with respect to EGFR-negative cases (median 1.52 OD, range 0.30–6.37 OD *vs* median 1.84, range 0.93–3.71 OD) (Table III).

**Figure 4** Distribution of p21 densitometric band intensities (expressed as optical density) in normal and neoplastic laryngeal tissues. Lines refer to the samples of normal mucosa and primary tumour derived from the same patient.

**Table II** Distribution of densitometric p21 band intensities according to histopathological and clinical characteristics of 43 primary laryngeal carcinomas

| | *No. of patients* | *Median value (OD)* | *Range (OD)* |
|---|---|---|---|
| Tumour location | | | |
| Supraglottic | 19 | 1.78 | 0.30–6.37 |
| Glottic | 11 | 1.26 | 0.40–3.71 |
| Transglottic | 13 | 2.11 | 1.03–5.47 |
| Stage | | | |
| I | 11 | 1.80 | 0.83–6.37 |
| II | 12 | 1.48 | 0.80–2.90 |
| III | 14 | 1.97 | 0.30–5.47 |
| IV | 6 | 2.40 | 1.26–4.95 |
| Lymph node involvement | | | |
| No | 28 | 1.55 | 0.83–5.47 |
| Yes | 15 | 1.99 | 0.30–6.37 |
| Histopathological grade | | | |
| G1 | 3 | 1.50 | 1.25–1.76 |
| G2 | 28 | 1.84 | 0.83–5.47 |
| G3 | 12 | 1.55 | 0.30–6.37 |

**Table III** Correlation of p21 oncoprotein expression with EGFR status in 43 primary laryngeal tumours

| | *No. of patients (%)* | *p21 expression (OD) Median* | *Range* | *P-value* |
|---|---|---|---|---|
| EGFR –[a] | 22 (51) | 1.84 | 0.93–3.71 | |
| EGFR + | 21 (49) | 1.52 | 0.30–6.37 | n.s. |

[a]8.00 fmol $mg^{-1}$ protein was used as an arbitrary cut-off to define EGFR status.

### Discussion

To our knowledge, this is the first study using the Western blotting technique to analyse p21 protein expression in normal laryngeal tissues and laryngeal tumours.

Varying levels of the p21 protein were observed in normal laryngeal specimens, as reported in other normal tissues (Walker *et al.*, 1988; Scambia *et al.*, 1993*a*,*b*), suggesting that p21 may be involved in normal cell functions and metabolism. We found that p21 levels were higher in neoplastic than in normal tissues when samples from the same patients were compared, thus confirming previous observations reported in breast (Spandidos *et al.*, 1984), colon (Gallick *et al.*, 1985), ovarian (Scambia *et al.*, 1993*a*) and endometrial (Scambia *et al.*, 1993*b*) tissues. These findings suggest that p21 overexpression may be associated with a malignant phenotype.

As far as laryngeal cancer is concerned, the presence of a wide range of p21 levels supports the hypothesis that overexpression of the p21 protein may play a role in the biology of laryngeal cancer cells, thus characterising more aggressive tumours. *ras* gene amplification has not been reported until now in laryngeal cancer (Merritt *et al.*, 1990; Sheng *et al.*, 1990). Therefore it is conceivable that transcriptional or post-transcriptional mechanisms are involved in p21 *ras* overexpression in laryngeal tumour cells. The MAb used in our study recognises both the abnormal and normal products of the three *ras* genes. The percentage of *ras* gene mutations has been reported to range from 4 to 14% in head and neck carcinomas (Sheng *et al.*, 1990; Anderson *et al.*, 1992; Irish, 1992), whereas there is no evidence of *ras* gene mutation in laryngeal tumours (Anderson *et al.*, 1992). It is conceivable that the role of altered p21 protein in laryngeal cancer is irrelevant and that the overexpression of each of the p21 gene products is responsible for *ras*-induced transformation. Despite the evidence that the p21 oncoprotein may be involved in mediating EGF mitogenic signals (Kamata & Feramisco, 1984), *ras* p21 expression was not related to EGFR levels as previously reported in gynaecological tumours (Scambia *et al.*, 1993*a*,*b*). It could be suggested that the EGF/EGFR system does not interact with *ras* or that the two systems are differentially regulated in laryngeal tumours.

The prognostic characterization of laryngeal cancer is still inadequate, and attempts should be made to identify new factors which could give further insight into tumour cell biology and the clinical evolution of this neoplasia.

It has been reported that p21 overexpression is associated with tumour progression and poor prognosis in breast cancer (Clair *et al.*, 1987). Moreover, our data indicate that p21 overexpression may have a negative prognostic value in ovarian cancer (Scambia *et al.*, 1993*a*).

Since the number of cases examined by us does not allow the evaluation of the possible role of p21 expression in the prognostic characterisation of laryngeal cancer patients, prospective studies are now in progress in our institute.

This study was supported by the special CNR project ACRO.G.F. is the recipient of a fellowship from the Italian Association for Cancer Research (AIRC).

### References

AMERICAN JOINT COMMITTEE ON CANCER (1993). *Handbook for Staging of Cancer*, 4th edn, pp. 57-61, J.B. Lippincott: Philadelphia.

ANDERSON, J.C., IRISH, J.C. & NIGAN, B.Y. (1992). Prevalence of ras oncogene mutation in head and neck carcinomas. *J. Otol.*, **21**, 321-326.

BARBACID, M. (1987). Ras genes. *Annu. Rev. Biochem.*, **56**, 779-827.

BATTAGLIA, F., SCAMBIA, G., ROSSI, S., BENEDETTI PANICI, P., BELLANTONE, R., POLIZZI, G., QUERZOLI, P., NEGRINI, R., IACOBELLI, S., CRUCITTI, F. & MANCUSO, S. (1988). Epidermal growth factor receptor in human breast cancer: correlation with steroid hormone receptors and axillary lymph node involvement. *Eur. J. Cancer Clin. Oncol.*, **24**, 1685-1690.

BOLTZ, E.M., KEFFORD, R.F., LEARY, J.A., HOUGHTON, C.R. & FRIEDLANDER, M.L. (1989). Amplification of c-ras-Ki oncogene in human ovarian tumours. *Int. J. Cancer*, **43**, 428-430.

BRADFORD, M.M. (1976). A rapid and sensitive method for the quantitation of microgram quantities of protein utilizing the principle of protein dye-binding. *Anal. Biochem.*, **72**, 248-255.

CARPENTER, G. (1987). Receptors for epidermal growth factor and other polypeptide mitogens. *Annu. Rev. Biochem.*, **56**, 881-884.

CLAIR, T., MILLER, W.R. & CHO-CHUNG, Y.S. (1987). Prognostic significance of the expression of ras protein with a molecular weight of 21,000 by human breast cancer. *Cancer Res.*, **47**, 5290-5293.

DE BORTOLI, M.E., ABOU-ISSA, H. & CHO-CHUNG, Y.S. (1985). Amplified expression of p21 ras protein in hormone dependent mammary carcinomas of humans and rodents. *Biochem. Biophys. Res. Commun.*, **127**, 699-706.

FURTH, M.E., DAVIS, L.J., FLEURDELYS, B. & SCOLNICK, E.M. (1982). Monoclonal antibodies to the p21 products of the transforming gene of the Harvey murine sarcoma virus and of the cellular ras gene family. *J. Virol.*, **43**, 294-305.

GALLICK, G.E., KURZROCK, R., KLOETZER, W.S., ARLINGHAUS, R.B. & GUTTERMAN, J.U. (1985). Expression of p21-ras in fresh primary and metastatic human colorectal tumors. *Proc. Natl Acad. Sci. USA*, **82**, 1795-1799.

GILMAN, A.G. (1984). G proteins and dual control of adenylate cyclase. *Cell*, **36**, 577-579.

HUGHES, D.A., FUKUI, Y. & YAMAMOTO, M. (1990). Homologous activators of ras in fission and budding yeast. *Nature*, **344**, 355-357.

IRISH, J.C. (1992). Oncogenes, molecular biology and the head and neck surgeon. *J. Otol.*, 3 (Suppl.), 2-20.

KAMATA, T. & FERAMISCO, J.R. (1984). Epidermal Growth Factor stimulates guanine nucleotide binding activity and phosphorylation of ras oncogene proteins. *Nature*, **310**, 644-649.

KURZROCK, R., GALLICK, G.E. & GUTTERMAN, J.U. (1986). Differential expression of ras p21 gene product among histological subtypes of fresh primary lung tumors. *Cancer Res.*, **46**, 1530-1534.

LONG, C.A., O'BRIEN, T.J., SANDERS, M., BARD, D.S. & QUIRK Jr J.G. (1988). Ras oncogene is expressed in adenocarcinoma of the endometrium, *Am. J. Obstet. Gynecol.*, **159**, 1512-1516.

MAURIZI, M., SCAMBIA, G., BENEDETTI PANICI, P., FERRANDINA, G., ALMADORI, G., PALUDETTI, G., DE VINCENZO, R., DISTEFANO, M., BRINCHI, D., CADONI, G. & MANCUSO, S. (1992). EGF receptor expression in primary squamous laryngeal cancer: correlation with clinico-pathological features and prognostic significance. *Int. J. Cancer*, **52**, 862-866.

MERRIT, W.D., WEISSLER, M.C., TURK, B.F. & GILMER, T.M. (1990). Oncogene amplification in squamous cell carcinoma of the head and neck. *Arch. Otolaryngol. Head Neck Surg.*, **116**, 1394-1398.

POWELL, J., ROBIN, P.E. (1983). Cancer of the head and neck: the present state. In *Head and Neck Cancer*. Rhys Evans, P., Robin, P.E. & Fielding, J.W.L. (eds). Castle House: Tunbridge Wells.

RODENBURG, C.J., KOELMA, I.A., NAP, M. & FLEUREN, G.J. (1988). Immunohistochemical detection of the ras oncogene product p21 in advanced ovarian cancer. *Arch. Pathol. Lab. Med.*, **112**, 151-154.

SATOH, T., ENDO, M., NAKAFUKU, M., AKIYAMA, T., YAMAMOTO, T. & KAZIRO, Y. (1990). Accumulation of p21 ras-GTP in response to stimulation with epidermal growth factor and oncogene products with tyrosine kinase activity. *Proc. Natl. Acad. Sci. USA*, **87**, 7926-7929.

SATOH, M., HATAKEYAMA, J., SASHIMA, M., SUZUKI, A. (1992). Immunohistochemical detection of ras p21 in oral squamous cell carcinomas. *Oral Surg. Oral Med. Oral Pathol.*, **74**, 469-472.

SCAMBIA, G., CATOZZI, L., BENEDETTI PANICI, P., FERRANDINA, G., CORONETTA, F., BAROZZI, R., BAIOCCHI, G., UCCELLI, L., PIFFANELLI, A. & MANCUSO, S. (1993*a*). Expression of ras oncogene p21 in normal and neoplastic ovarian tissues: Correlation with histopathologic features and receptors for estrogen, progesterone, and epidermal growth factor. *Am. J. Obstet. Gynecol.*, **168**, 71-77.

SCAMBIA, G., CATOZZI, L., BENEDETTI PANICI, P., FERRANDINA, G., BATTAGLIA, F., GIOVANNINI, G., DISTEFANO, M., PELLIZZOLA, D., PIFFANELLI, A. & MANCUSO, S. (1993*b*). Expression of ras p21 oncoprotein in normal and neoplastic human endometrium. *Gynecol. Oncol.*, **50**, 339-346.

SHENG, Z.M., BARROIS, M., KLIJANIENKO, J., MICHEAU, C., RICHARD, J.M. & RIOU, G. (1990). Analysis of the c-Ha-ras-1 gene for deletion, mutation, amplification and expression in lymph node metastases of human head and neck carcinomas. *Br. J. Cancer*, **62**, 398-404.

SMIT, V.T.H.B.M., BOOT, A.J.M., SMITS, A.M.M., FLEUREN, G.J., CORNELISSE, C.J. & BOS, J.L. (1988). K-ras codon 12 mutations occur very frequently in pancreatic adenocarcinomas. *Nucleic Acids Res.*, **16**, 7773-7782.

SPANDIDOS, D.A. (1987). Oncogene activation in malignant transformation: a study of H-ras in human breast cancer. *Anticancer Res.*, **7**, 991-996.

SPANDIDOS, D.A. & KERR, I. (1984). Elevated expression of the human ras oncogene family in premalignant and malignant tumors of the colorectum. *Br. J. Cancer*, **49**, 681-688.

TANAKA, T., SLAMON, D.J., BATTIFORA, H., CLINE, M.J. (1986). Expression of p21 ras oncoproteins in human cancers. *Cancer Res.*, **46**, 1465-1470.

VANT'T VEER, L.J., HERMENS, R., VAN DEN BERG-BAKKER, L.A.M., CHENG, N.C., FLEUREN, G.J., BOS, J.L., CLETON, F.J. & SCHRIER, P.I. (1988). Ras oncogene activation in human ovarian carcinomas. *Oncogene*, **2**, 157-165.

VIOLA, M.V., FROMOVITZ, F., ORAVEZ, S., DEB, S. & SCHLOM, J. (1985). Ras oncogene p21 expression is increased in premalignant lesions and high grade bladder carcinoma. *J. Exp. Med.*, **161**, 1213-1218.

VOGELSTEIN, B., FEARON, E.R., HAMILTON, S.R., KERN, S.E., PREISINGER, A.C., LEPPERT, M., NAKAMURA, Y., WHITE, R., SMITS, A.M.M. & BOS, J.L. (1988). Genetic alterations during colorectal tumor development. *N. Engl. J. Med.*, **31**, 525-532.

WALKER, R.A. & WILKINSON, N. (1988). P21 ras protein expression in benign and malignant human breast. *J. Pathol.*, **156**, 147-153.